# Selenium nanoparticles (SeNPs) have potent antitumor activity against prostate cancer cells through the upregulation of miR-16

**DOI:** 10.1186/s12957-020-01850-7

**Published:** 2020-05-01

**Authors:** Guolong Liao, Jiani Tang, Di Wang, Haoru Zuo, Qi Zhang, Ying Liu, Haiyun Xiong

**Affiliations:** 1 Department of Urology, The Seventh Affiliated Hospital of Sun Yat-Sen University, Shenzhen, 518107 China; 2 Department of Clinical Laboratory, PLA 309 Hospital, Beijing, China; 3 Department of Surgery Anesthesia Center, the Seventh Affiliated Hospital of Sun Yat-Sen University, Shenzhen, 518107 China

**Keywords:** Selenium nanoparticles, Prostate cancer, Cell cycle, Apoptosis, MicroRNA

## Abstract

**Objectives:**

This research aimed to examine the antitumor mechanisms of selenium nanoparticles (SeNPs) specifically against prostate cancers.

**Methods:**

The antitumor activities of SeNPs against cancer cells were determined via MTT assay. The cell cycle was determined by detecting the DNA content, and apoptosis was determined via annexin V-Fluos staining kit. The microRNA expressions in cancer cells were analyzed via microarray and qRT-PCR. The potential targets of miR-16 were identified via luciferase analysis and mRNA expression determination. miR-16 functions in cancer cells were explored via the transient transfection of miR-16 mimic or inhibitor.

**Results:**

SeNPs were most potent in prostate cancer cells, regardless of whether or not they were androgen-dependent. Furthermore, SeNP stimulation can induce cell cycle arrest and the apoptosis enhancement of prostate cancer cells. Microarray and molecular mechanism studies demonstrated that miR-16 could directly target cyclin D1 and BCL-2 to mediate SeNP apoptosis enhancement. Results show that the serum selenium levels positively correlate with miR-16 expressions, and they correlate with the overall and disease-free survival rates.

**Conclusion:**

These results signify the cytotoxic potential of SeNPs in prostate cancer treatment.

## Introduction

Prostate cancer (CaP) is the second most prevalent cancer globally [1]. Prostate cancer is also the fifth most common cancer-related cause of male death. Early-stage CaP requires androgens to grow, and if it is caught before progressing further, then it often responds well to androgen deficiency therapy [2]. However, advanced CaP can transform from androgen-dependent prostate cancer (ADPC) into androgen-independent prostate cancer (AIPC) [3]. Currently, no effective treatment exists for AIPC. Therefore, developing effective drugs for CaP is an urgent need, especially for AIPC, which could reduce the mortality and improve the quality of life of CaP patients.

Selenium has been shown to have antitumor properties, and selenium supplements have been used in individual anticancer therapies [4]. Owing to their minimal risk relative to selenium on its own, selenium nanoparticles (SeNPs) have had significant applications in medical diagnosis over the last decade, and thus, they have been widely used as dietary supplements and antioxidants [5]. In particular, SeNPs have occasionally been reported to exhibit antitumor effects against several tumor types, including glioma, lung cancer, and breast cancer [4, 5].

The promotion of apoptosis of cancer cells by SeNPs is generally believed to be an important mechanism in their inhibition of malignant tumors [4]. SeNPs have been found to regulate key apoptotic proteins, such as the caspase family, p53, and ROS [6]. However, these sporadic findings do not fully explain the antitumor functions of SeNPs. In particular, the question of whether or not microRNAs (miRNA), which play an important role in tumorigenesis and development, are regulated by SeNPs requires further study.

In the previous work of the authors, SeNPs were synthesized using new microbial technology and characterized via UV-Vis absorption spectroscopy [6]. In those studies, SeNPs were found to play an important role in inhibiting lung cancer cells but had little effect on normal cells. Similarly, SeNPs have been suggested to have potent cytotoxicity against tumor cells, but not against normal cells, in studies of cervical carcinoma [7], hepatocarcinoma [8], and colorectal cancer [9]. Therefore, plans were made to test for the inhibitory effects of SeNPs on other cancer cells and to explore their molecular mechanisms. Preliminary experiments confirmed that SeNPs are significantly active on several cancer cell lines and that their activity on CaP cells is stronger than on other cancer cells. This indicates that SeNPs might be most effective in CaP and that SeNPs lead to cell cycle arrest and apoptosis enhancement in various CaP cell lines and primary isolated tumor cells. Molecular biological experiments suggest that SeNP-induced apoptosis enhancement is achieved in part by miR-16 upregulation.

## Material and methods

### Preparation and characterization of SeNPs

The methods of the SeNPs biosynthesis and characterization were described in our previous work [6]. Briefly, a 250-ml Erlenmeyer flask was used to harvest the bacterial seed culture in 100 ml N-broth growth medium and incubated at 30 °C for about 24 h. Three millimolars of sodium selenite (0.0518 g) and activated *Escherichia coli* (*E. coli*) culture (1 ml) were inoculated into 100 ml sterilized N-broth medium, placed in an incubator at 30 °C for 48 h at 150 rpm. UV-visible (HACH, DR 5000, USA) spectrophotometer was employed to analyze the samples. The mixture was centrifuged at 12,000 rpm for 10 min to isolate the particles from the reaction mixture and then washed thrice using acetone and distilled water. For control experiment, active *E. coli* culture was autoclaved for 15 min and cultivated into 100 ml sterilized N-broth medium with 3 mM (0.0518 g) sodium selenite under similar conditions as described above. For characterization of SeNPs, transmission electron microscopy (TEM; Zeiss-EM10C) was employed to study the dimension and morphology of the synthesized SeNPs at a raising voltage of 80 kv. Ultraviolet visible (UV-vis) spectrophotometer at a resolution of 2 nm was used to record the absorption spectra at a wave length range of 200–1000 nm. For the elemental analysis of the product, energy-dispersive X-ray analysis (EDAX) analyzer (JSM-7600F, JEOL, Japan) was used. XRD analysis (Xpert pro, PANalytical, Holland) was performed to investigate the crystalline phase of synthesized SeNPs.

### Cell line culture

Eight tumor cell lines, including Colon-26 (colon cancer), LNCaP (CaP), HepG2 (liver cancer), Hela (cervical cancer), A549 (NSCLC), MCF7 (breast cancer), A498 (renal cancer), and WM-115 (melanoma), which were purchased from ATCC (Manassas, VA, USA), were used in the preliminary screening of the anticancer activity of SeNPs. For further study on the antitumor activity of SeNPs against CaP, LNCaP (ADPC), PC-3 (AIPC), C4-2 (AIPC), LNCaP-A (AIPC), PWR-1E (normal prostate), and RWPE-1 (normal prostate), also purchased from ATCC, were included. For the cell culture, 10% fetal bovine serum (FBS) (Invitrogen, Grand Island, NY, USA), 100 U/ml streptomycin, and 100 U/ml penicillin (Hyclone laboratories Inc., South, UT, USA) were added to Roswell Park Memorial Institute (RPMI-1640) to prepare the cell culture medium. The culture environment was 5% carbon dioxide and 37 °C with humidified air in an incubator (Thermo Fisher, Waltham, MA, USA).

### Clinical samples

This study was conducted in the urology department of the Seventh Affiliated Hospital of Sun Yat-Sen University. One hundred seventeen inpatients with CaP and 33 healthy controls were enrolled in this study. Serum samples from all patients, and their matched controls, were collected during morning admission (on an empty stomach). Patients treated for CaP before their serum sample extraction were excluded from this study. ADPC patients or AIPC patients were considered by referring to the documentary reporting criterion [10]. Sixty-two patients were allocated to the AIPC group and while others were allocated to the ADPC cases. The serum selenium level was determined via graphite Atomic Absorption Spectrophotometer (AAS) as described in the previous report [11].

### Isolation of the primary CaP cells

CaP tissues and adjacent normal tissues were obtained from 117 CaP patients via surgery, then primary cultured cells were extracted from them according to previous reports [12]. The CaP tissues and adjacent normal tissues were washed with 95~100% (v/v) ethanol and PBS (0.01Μ, ρΗ7.4), then they were placed in a sterile Petri dish containing pre-cooled PBS. Residual fat was removed with anatomical forceps and scissors. Next, 0.25% trypsin or 2000 U/ml collagenase was added into the dish, digestion in a 37 °C water bath for 30 min was conducted, the digestive juice was removed, washing was done three times with washing solution, the medium was washed once, suspended with complete medium, and a straw was used to blow and disperse into the cell suspension. Then, the mixture was centrifuged, and the pellet was re-suspended in PBS containing 0.25% trypsin/1 mM EDTA for 30 min at 37 °C. The trypsin is neutralized with 10% FCS, and the cells were washed twice in PBS. The cells are subsequently passed through a cell strainer to ensure single-cell suspension (BD Falcon, NJ). The cells were cultured via RPMI-1640 medium.

### MTT assay

The antitumor activities of SeNPs against different types of cancer cells were determined via MTT (3-(4,5-dimethylthiazole-2-yl)-2,5-diphenyltetrazolium bromide). After counting the cells to be measured, the cells were inoculated into 96-well plates with 10,000 cells per well for 24 h, then the cells were treated with SeNPs at certain concentrations for 24 h. Subsequently, the cells were treated with MTT solution for 3 h, DMSO was added, then the cells were placed for 15 min. Finally, an ELISA microplate reader (DYNEX, USA) was used to measure absorbance at 570 nm (OD value). The standard MTT assay curve was established via the following process: Cells were counted then inoculated into clear cell culture plates at seven concentrations: 1000/well, 2000/well, 4000/well, 8000/well, 16000/well, 32000/well, and 64000/well. They were incubated with MTT reagent at 37 °C for 3 h. After incubation, the cells were treated with DMSO at room temperature for 15 min. Absorbance was measured at OD = 570 nm, and a standard curve was drawn based on the correlation between OD values and cell numbers. The cell number was calculated via the corresponding OD value through the standard curve. The SeNP inhibition rate was calculated via the following equation: inhibition rate = (cell number(control) − cell number(SeNP treatment))/cell number(control) × 100%.

### Cell cycle analysis

Cells were cultured in a serum starvation medium at the G0 stage for 3 days, and then, the medium was replaced by a medium containing 10% FBS for the culture. Cell cycle progression is monitored by detecting the DNA content [13]. Cells were washed twice with PBS, trypsinized, and then fixed in 70% methanol for 2 h at − 20 °C. Subsequently, cells were precipitated (5 min of centrifugation at 500×*g* at 4 °C in a Sigma 6 K15 centrifuge), washed with PBS, and re-suspended in 1 ml of PBS containing 40 U of RNase A per ml and 40 μg of propidium iodide per milliliter. After incubating for 30 min at 37 °C, DNA flow cytometric analysis was performed with an EPICS XL-MCL flow cytometer (Coulter). Multicycle AV software (Phoenix Flow Systems) was used for quantification.

### Cell apoptosis analysis

Apoptotic cells were detected via annexin V-Fluos staining kit (Roche-Boehringer). Cells (1 × 10^6^)/well were plated in 6-well plates for 24 h then treated with SeNPs at specific concentrations for an additional 24 h in a 5% CO_2_ humidified atmosphere at 37 °C. At the end of the incubation period, the treated cells and controls were harvested and incubated with Annexin V and PI for 15 min before being analyzed on a flow cytometer with 488 nm excitation, 515 nm for Annexin V detection, and a filter with the wavelengths above 600 nm for PI detection.

### Determination of Caspase-3 activity

The flourometric method was used to evaluate the Caspase-3 activity according to the previous report [6]. Twelve-well plates were seeded with cells and incubated for 24 h. Later, the cells were treated with SeNPs and incubated for 24 h. A lysis buffer was used for cell lysis after harvesting, then the cells were incubated for 1 h in ice. Caspase-3 substrates (Ac-DEVD-AMC) were used to determine the Caspase activity by measuring fluorescence intensity at an excitation wavelength of 380 nm and 460 nm as an emission wavelength.

### Reverse transcription-quantitative polymerase chain reaction (RT-qPCR) assay

TRIzol reagent (Invitrogen, Grand Island, NY, USA) was used to obtain the total RNA from cells. Subsequently, qRT-PCR for mRNA was performed via the PrimeScript RT-PCR kit (Takara, Bio, Inc., Shiga, Japan) on an IQ5 fluorescence quantitative PCR detector (Bio-Rad, Hercules, CA, USA). Primer sequences for all the targets and β-actin are described in the following way: Primer for β-actin: forward 5′-AAGGAAGCTTGGCGTTGTGA-3′; reverse: 5′-GAGAGGTGAGGAGTCTTATG-3′. Primer for P21: forward 5′-TAGCAGCGGAACAAGGAG-3′; reverse: 5′-AAACGGGAACCAGGACAC-3′. Primer for cyclin D1: forward 5′-GTCTTCCCGCTGGCCATGAACTAC-3′; reverse: 5′-GGAAGCGTGTGAGGCGGTAGTAGG-3′. Primer for BAX: forward 5′-TCCACCAAGAAGCTGAGCGAG-3′; reverse: 5′-GTCCAGCCCATGATGGTTCT-3′. Primer for BCL-2: forward 5′-TTCTTTGAGTTCGGTGGGGTC-3′; reverse: 5′-TGCATATTTGTTTGGGGCAGG-3′. The following parameters were used for the reverse transcription reaction: 65 °C for 5 min, 37 °C for 15 min, and 98 °C for 5 min. The following parameters were used for the subsequent PCR reaction: 95 °C for 30 s and then 40 cycles of 95 °C for 5 s, 60 °C for 5 s, and 72 °C for 30 s.

qRT-PCR for microRNAs was conducted using TaqMan miRNA assays (Ambion; Thermo Fisher Scientific, Inc.) and has-miR-16 RT-PCR primer set (Abbexa, Inc., Cambridge, UK). Reverse transcription reactions were performed via TAQMAN® microRNA RT kit (Ambion; Thermo Fisher Scientific, Inc.) under the following conditions: 16 °C for 30 min, 42 °C for 30 min, and 84 °C for 5 min. PCR reactions were conducted under the following conditions: 95 °C for 2 min followed by 40 cycles of 95 °C for 15 s and 60 °C for 30 s. U6 small nuclear RNA was used as an endogenous control for data normalization.

### Microarray analysis of miRNA expression

Microarray detection was carried out via the Agilent Human miRNA microarray platform (Agilent Technologies) by Shanghai Biochip Co., Ltd. (Shanghai, China). The Agilent human microarray platform (Agilent technology) and its microarray scanner were used for microarray analysis and microarray slide scanning. The scanned image was analyzed via Feature Extraction software 10.7.1.1 (Agilent). Genespring 12.0 (Agilent) was used to normalize the raw data.

### Dual luciferase assay for BCL-2 and cyclin D1 targets identification

Double-stranded DNA fragments containing the potential miR-16 binding site sequences of BCL-2 and cyclin D1 genes were prepared. Then, the DNA fragments were introduced into the pGL3-promoter vector (Promega, Madison, WI, USA) to construct pGL3-wide type-BCL-2 and pGL3-wide type-cyclin D1 plasmid. Both pGL3-wide type-BCL-2 and pGL3-wide type-cyclin D1 had GCUGCU sequence in the binding site for miR-16. By using a site-directed mutagenesis kit (Stratagene), this GCUGCU sequence was replaced with GAAAAU to construct pGL3-mutant-BCL-2 and pGL3-mutant-cyclin D1. The recombinant plasmid constructions were identified via DNA sequencing.

### Statistical analyses

By using SPSS software version 17.0 (SPSS Company, Chicago, Illinois, USA), the statistical differences between the experimental group and the control group were determined via the t-test of the double-tailed Student. All experiments in this study were repeated three times, and data are represented as the mean ± standard deviation. If the *p* value is less than 0.05, a statistical difference between the two comparison groups will be set.

## Results

### In vitro antitumor activity of SeNPs against eight tumor cell lines

**Fig. 1:**
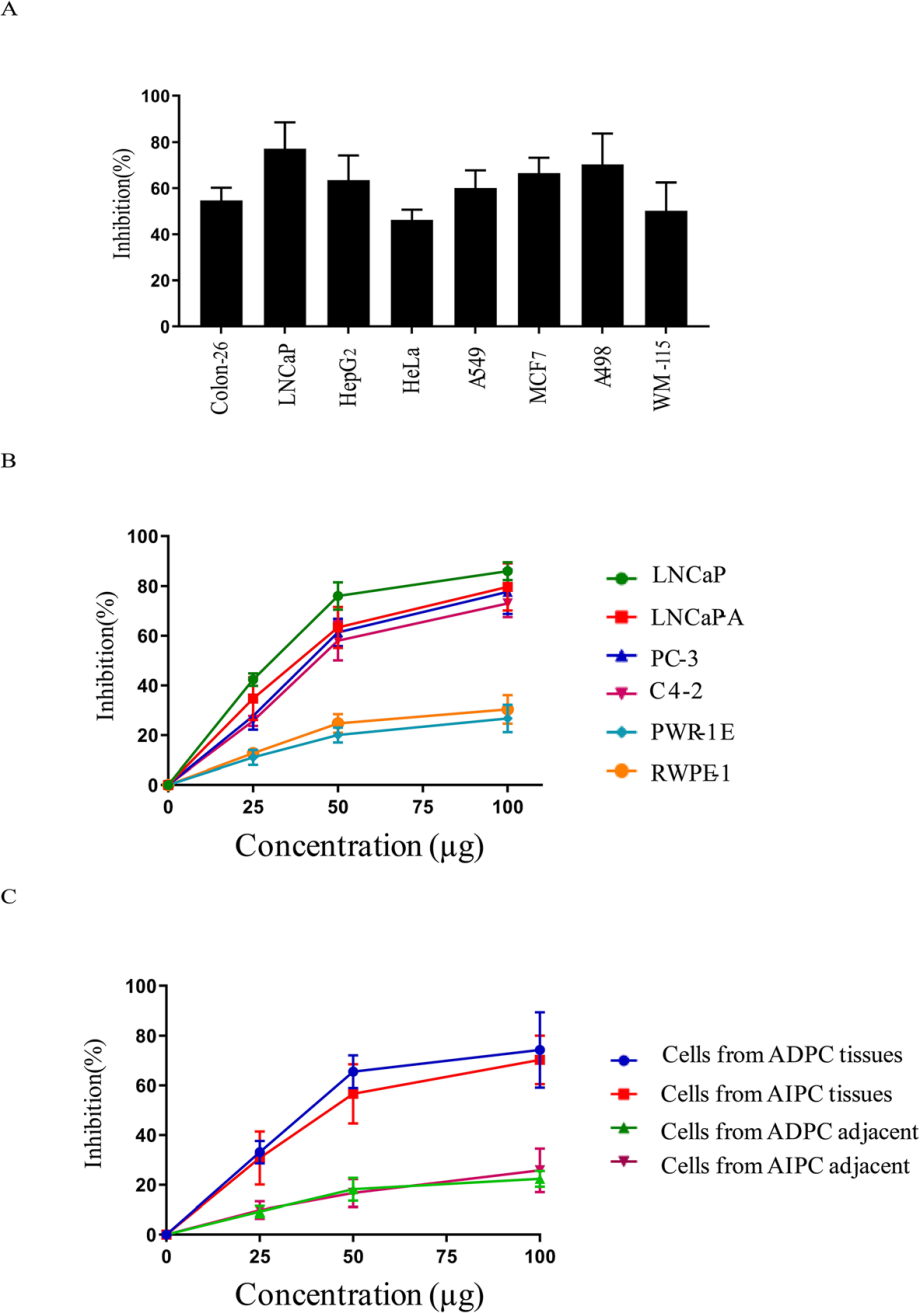
SeNPs has potent antitumor activities against different cancer cells in vitro. **a** Inhibition percentages expressed by the SeNPs against eight cancer cell line at a single-dose of 50 μg. **b** Inhibition percentages expressed by the SeNPs against four prostate cancer cell lines and two normal prostate cells at dose ranges from 25 to 50 μg. **c** Inhibition percentages expressed by the SeNPs against patients-derived prostate cancer cells at dose ranges from 25 to 100 μg. The %inhibition values were calculated by subtracting the growth percentages from 100.

The essential properties of nanoparticles, including element composition, size, shape composition, and surface structure, can affect the anticancer activity of nanoparticles [14]. The average SeNP crystalline size used in this study was calculated as 88.89 nm via X-ray diffraction, which was identified as a hexagonal ring structure with a diffraction ring pattern [6]. The antitumor activities of this kind of SeNPs against eight cancer cell lines were initially tested. Figure 1a shows the inhibition percentages caused by a single 50 μg dose of SeNPs. The inhibition percentages of SeNPs on cancer cells ranged from 46.3 ± 4.4% to 77.2 ± 11.4%. The most obvious inhibitory effect was observed in CaP cells.

### In vitro antitumor activity of SeNPs against the CaP cells

Subsequently, the anticancer activity of SeNPs at different concentrations was measured against several CaP cell lines, including ADPC and AIPC. These results show that the treatment of LNCaP cells with 25 μg/ml, 50 μg/ml, and 100 μg/ml each led to inhibition percentages of 42.3 ± 2.5%, 77.2 ± 11.4%, and 82.7 ± 10.5%. AIPC cell inhibition was slightly lower, especially with a low concentration of SeNPs. SeNPs had no significant effect on normal healthy cells (Fig. 1b). For each CaP patient, primary cells were extracted from CaP tissues and tumor-adjacent tissues then the inhibitory effect of SeNPs against these cells was measured. Figure 1c shows that SeNPs have potent toxicity against both ADPC and AIPC the primary isolated CaP cells of ADPC and AIPC. Collectively, these results indicate that SeNPs have a strong cytotoxic effect against CaP cells.

### SeNP treatment leads to cell cycle arrest and enhanced apoptosis in CaP cells

**Fig. 2:**
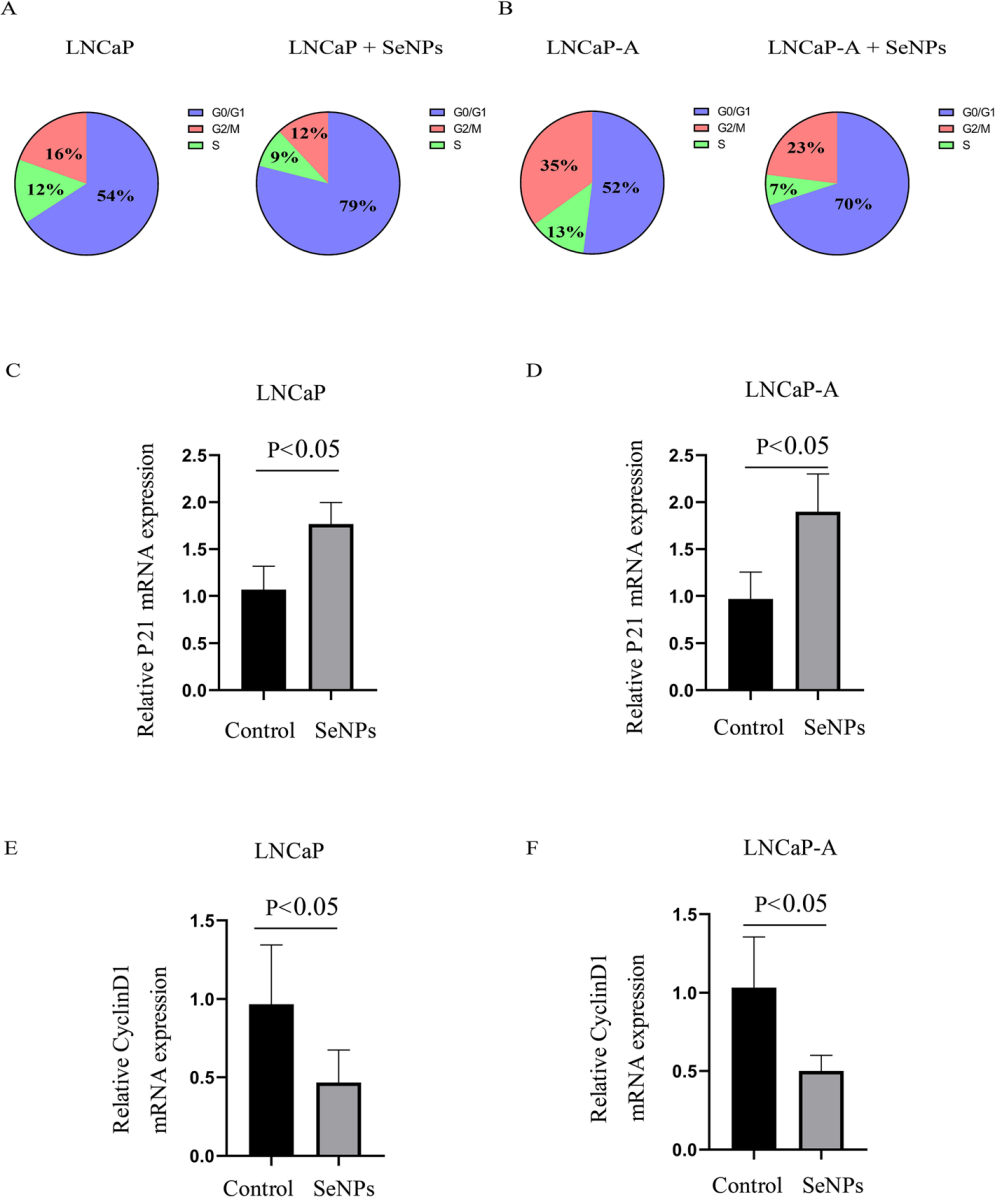
SeNP treatment lead to cell cycle arrest in LNCaP and LNCaP-A cells. **a**, **b** Cell cycle analysis in **a** LNCaP and **b** LNCaP-A after SeNP treatment. **c**, **d** Expression of p21 (CDKN1A, **c**) in **c** LNCaP and **d** LNCaP-A after SeNP treatment. **e**, **f** Expression of cyclin D1 in **a** LNCaP and **b** LNCaP-A after SeNP treatment. SeNP dose: 50 μg

Next, the anti-CaP mechanism of SeNPs in LNCaP and LNCaP-A cells was studied. When compared with the control group, the cell cycle arrest rate in LNCaP and LNCaP-A cells was significantly increased after treatment with 50 ug/ml SeNPs (Fig. 2a, b). Cell cycle arrest has been reported to be induced by p21 and inhibited by cyclin D1 [12]. As expected, the p21 mRNA expression was significantly increased in SeNP-treated LNCaP and LNCaP-A cells (Fig. 2c, d), while the cyclin D1 expression was decreased (Fig. 2e, f).

**Fig. 3:**
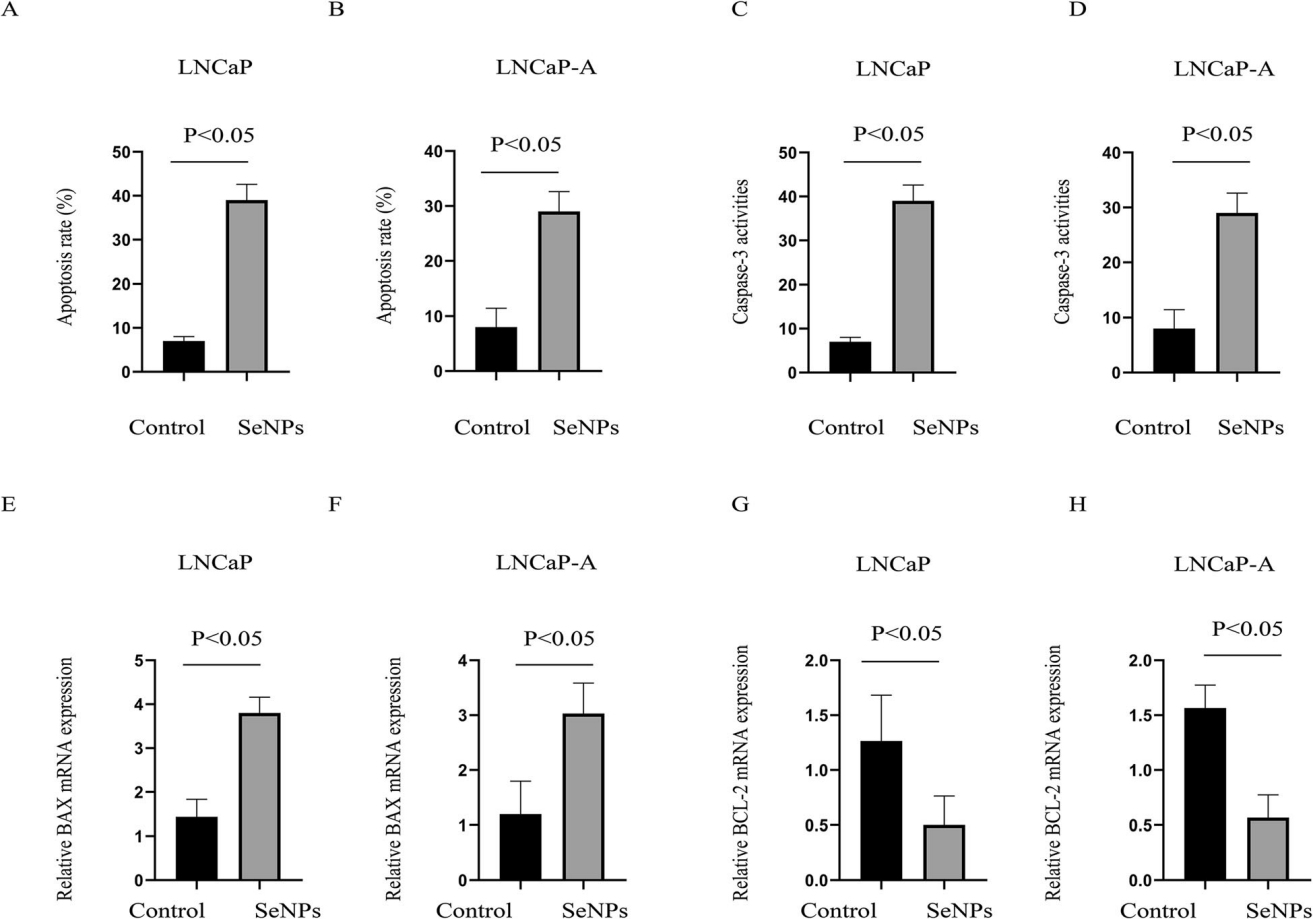
SeNP treatment lead to cell apoptosis enhancement in LNCaP and LNCaP-A cells. **a**, **b** Cell apoptosis analysis in **a** LNCaP and **b** LNCaP-A after SeNP treatment. **c**, **d** Cell casepase-3 activities analysis in **c** LNCaP and **d** LNCaP-A after SeNP treatment. **e**, **f** Expression of Bax in **e** LNCaP and **f** LNCaP-A after SeNP treatment. **g**, **h** Expression of BCL-2 in **a** LNCaP and **b** LNCaP-A after SeNP treatment. SeNP dose: 50 μg

Furthermore, the SeNP treatment was found to significantly enhance both the apoptosis rate (Fig. 3a, b) and caspase 3 activity (Fig. 3c, d) in LNCaP and LNCaP-A cells. In addition, 24 h after SeNP treatment, the BAX mRNA levels were upregulated (pro-apoptotic, Fig. 3e, f), and the BCL-2 expression was significantly downregulated (anti-apoptotic, Fig. 3g, h). These results lead to the conclusion that SeNP treatment can cause cell cycle arrest and apoptosis enhancement.

### Global changes in the expression of miRNAs in CaP cells during SeNP treatment

**Fig. 4:**
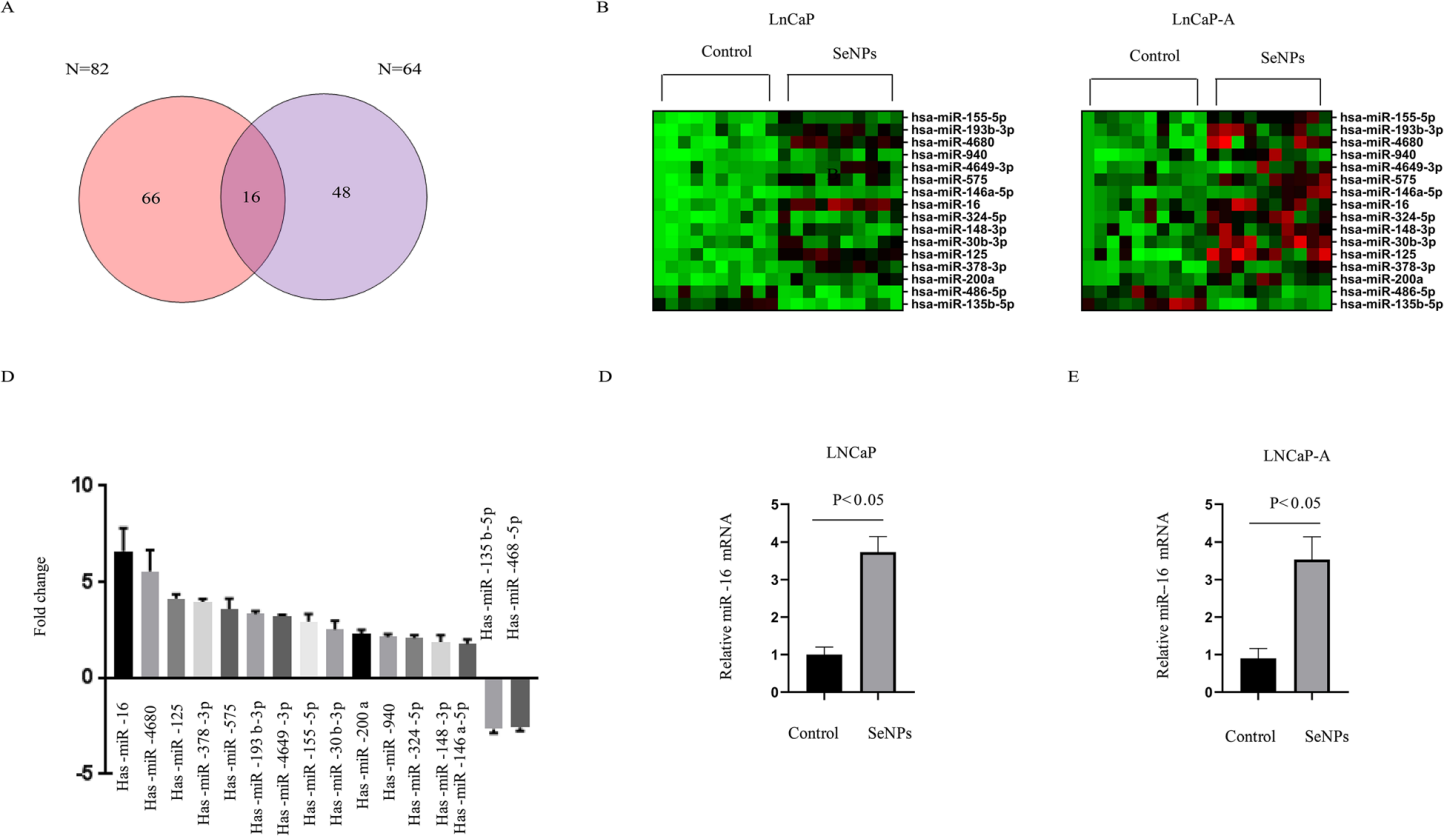
Microarray analyses reveal a role of SeNPs in microRNA expressions. **a** The Venn diagrams depict the number of genes regulated by SeNP treatment in LnCaP and LnCaP-A cells identified by microarray analysis. Overlapped regions represent the number of genes regulated by SeNP treatment in both LnCaP and LnCaP-A cells. **b** Heatmap clustering analysis of microRNA expression profile of the overlapped targets in **a** in both LnCaP and LnCaP-A cells. Each column represents the microRNAs expression profile. **c** Fold change of top differentially expressed genes by SeNP treatment. **d**, **e** Confirmation of miR-16 expression in **d** LNCaP and **e** LNCaP-A after SeNP treatment by qRT-PCR analysis

SeNP-treated LNCaP and LNCaP-A cells were used to investigate global changes in the miRNA expressions. With a cut-off line of > 1.5 fold change and *p* ≤ 0.05 (identified by one-way ANOVA), 212 genes in LnCaP and 244 genes in LnCaP-A were differentially expressed after SeNP treatment. Venn analysis showed that a total of 16 differentially regulated genes overlapped in SeNP-treated LNCaP and LNCaP-A cells (Fig. 4a). Figure 4b shows the clustering of these genes. Among these genes, miR-16 showed the largest expression change in a microarray assay (Fig. 4c). The miR-16 expression profile in SeNP-treated LNCaP and LNCaP-A cells was further validated via qPCR analysis (Fig. 4d, e).

### SeNP treatment enhances cell apoptosis in CaP cells via miR-16 upregulation

**Fig. 5:**
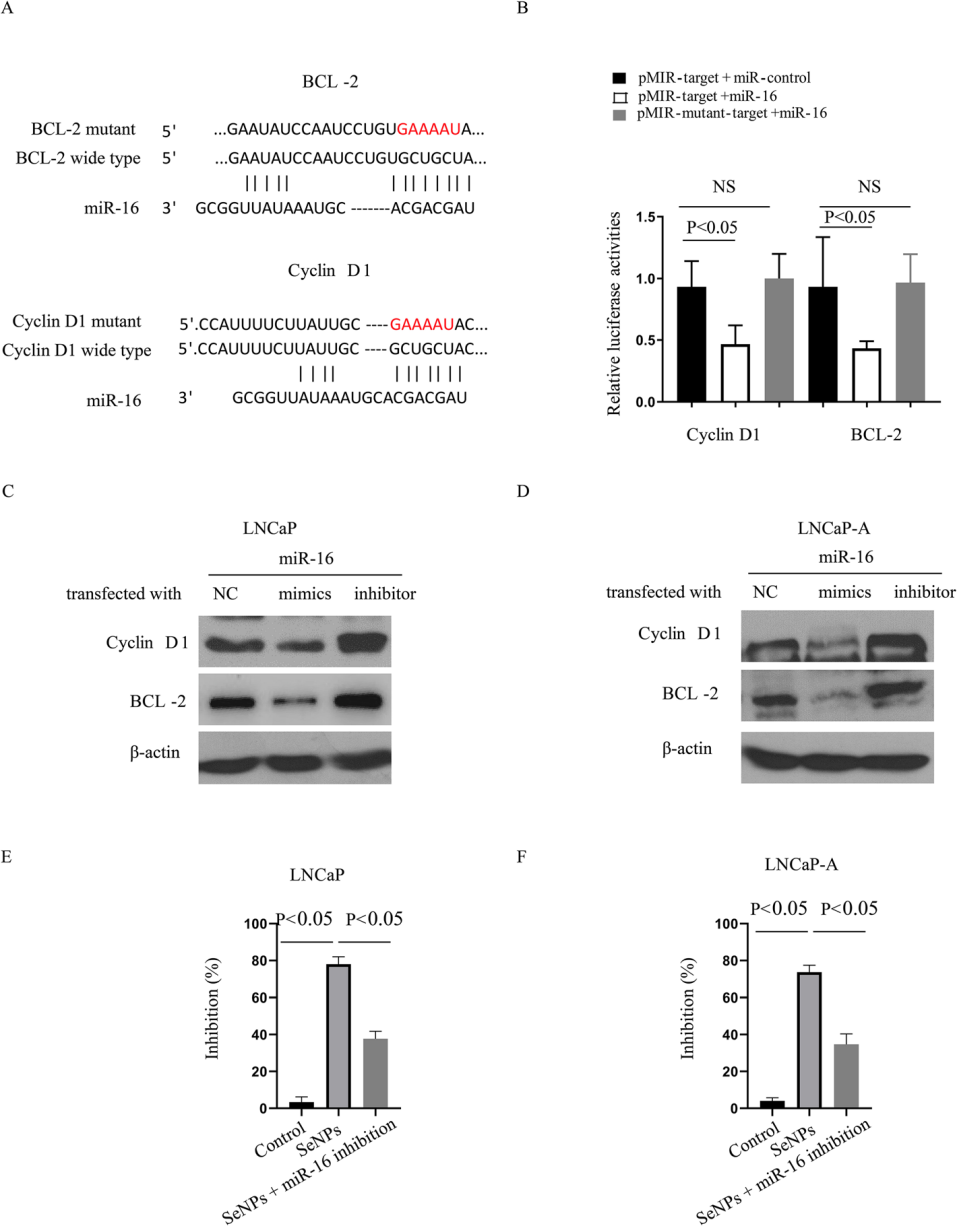
SeNP treatment enhance the cell apoptosis of the prostate cancer cells through miR-16 upregulation. **a** Sequence alignment of miR-16 and its target sites in 3′ untranslated regions of BCL-2 and cyclin D1. BCL-2 and cyclin D1 mutant site was shown in red. **b** HEK293 cells were transiently co-transfected with luciferase report vectors, and either miR-16 mimics or scrambled miR-control. Luciferase activities were normalized to the activity of Renilla luciferase. **c** LNCaP and **d** LNCaP-A were transient transfected with miR-16 inhibitor, together with SeNP treatment, and the protein expressions of BCL-2 and cyclin D1 were determined. **e** LNCaP and **f** LNCaP-A were transient transfected with miR-16 inhibitor, together with SeNP treatment, and the inhibition percentages expressed by the SeNPs were determined. The %inhibition values were calculated by subtracting the growth percentages from 100. SeNP dose: 50 μg

TargetScan analyses (http://www.targetscan.org/) were used to find that cyclin D1 and BCL-2, key proteins in the proliferation and apoptosis of cancer cells, might bind with miR-16 at a GCUGCU sequence site (Fig. 5a). This prediction was confirmed using a luciferase report system, which showed that miR-16 mimics significantly inhibited luciferase activity in HEK293 cells containing the wild-type 3′UTR of BCL-2 or cyclin D1. However, luciferase activity was not affected in cells with the mutant 3′UTR of BCL-2 or cyclin D1 (Fig. 5b). Moreover, miR-16 mimics significantly decreased the protein expression of BCL-2 and cyclin D1 in LNCaP and LNCaP-A cells (Fig. 5c, d), indicating that miR-16 upregulation might be essential to mediating SeNP antitumor activity. To investigate this point, LNCaP and LNCaP-A cells were transiently transfected with a miR-16 inhibitor in conjunction with a treatment of 50 μg/ml SeNPs. Figures 5e and f show the use of an miR-16 inhibitor sharply reversed the impacts of SeNPs on the apoptosis rate. These findings suggest that SeNP treatment increases apoptosis rates by enhancing miR-16 expression.

### Selenium content in CaP patients is correlated with clinical prognosis

**Fig. 6:**
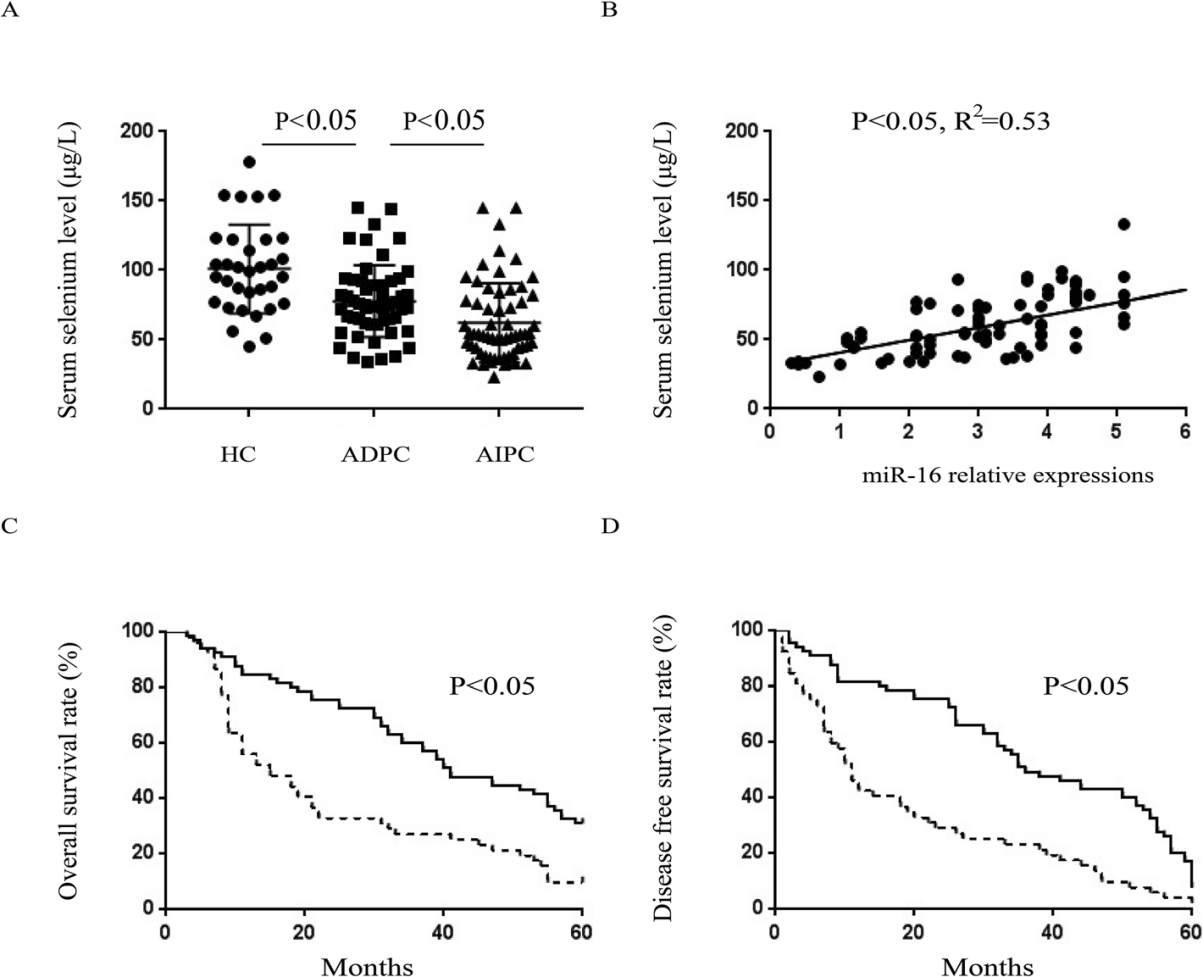
Serum selenium content in prostate cancer patients is correlated with clinical prognosis. **a** Comparing the serum selenium content among AIPC, ADPC, and healthy control groups. **b** Determination of the correlation between serum selenium content and serum miR-16 expressions. The **c** overall survival and **d** disease-free survival of prostate cancer patients with different serum selenium level. Solid line: serum selenium level high group; dotted line: serum selenium level low group

Next, whether or not the role of selenium in patients was consistent with the results needed to be further verified. In the entire patient cohort, the serum selenium concentration for CaP patients before treatment was significantly lower than that for healthy controls (Fig. 6a). A positive correlation existed between selenium content and serum miR-16 expression, which was in agreement with the cytological experiment results (Fig. 6b). Subsequently, all CaP subjects were categorized into either high or low serum selenium groups according to whether or not they were above or below the mean value and were subsequently followed up (57.1 μg/L). Kaplan–Meier analysis showed that patients in the high selenium cohort had longer overall survival (OS) time and disease-free survival (DSF) time (Fig. 6c, d). In summary, the data from these studies suggest that higher selenium concentrations in CaP patients before treatment correlates with better prognoses.

## Discussion

SeNPs have been suggested to have antitumor activity, and many studies have shown that they have high specific toxicity for cancer cells [15–17]. However, to date, whether or not SeNPs have different inhibitory potential against different types of tumors has not been made clear. This study explored SeNP toxicity on eight cancer cell lines, and SeNPs were found to have the strongest effect on CaP. Several previous reports have suggested that SeNPs exhibit strong inhibitory effects on CaP [18, 19]. For example, Sonkusre et al. reported that SeNPs have potent toxicity against PC-3 cancer cells [18]. Kong et al. demonstrated that SeNPs could suppress the growth of LNCaP cancer cells [19]. However, these studies primarily focused on a single CaP cell line. Furthermore, the clinical data on SeNP activity is sparse. This work appears to be a very early exploration of the antitumor effect of SeNPs against patient-derived CaP cells in vitro. SeNP inhibitory potential was slightly less for primary isolated CaP cells, as compared with CaP cell lines. This is possibly due to the existence of non-neoplastic cells in primary isolated cells. However, these experiments generally confirm that SeNPs have a significant inhibitory effect on patient-derived CaP cells, regardless of whether or not they were androgen-dependent. Meanwhile, SeNP toxicity on normal prostate cells is relatively low. This data indicates that SeNPs could represent a promising anti-CaP drug.

The molecular mechanism by which SeNPs induce tumor suppression is not yet fully understood. SeNPs are generally believed to be able to trigger tumor cell apoptosis by enhancing cellular uptake and blocking reactive oxygen species (ROS) [20]. Huang et al. have shown that SeNPs can stimulate cancer cell autophagy, thereby playing an anticancer role [21]. The report by Sonkusre showed that SeNPs induced TNF upregulation, which can activate cancer cell necrosis [18]. Vekariya et al. showed that SeNPs inhibited cell growth and the synthesis of DNA, RNA, and proteins, suggesting that SeNPs may alter the expression of a large number of functional molecules including non-coding RNA [17].

Additional evidence regarding the novel role of miRNAs in the cell cycle and apoptosis of cancer cells has been found recently. Most miRNA functions through the suppression of their target genes [22–24]. Unexpectedly, few reports have come up describing the relationship between SeNP treatment and miRNA expression. The highlight of this study was that using microarray technology, a series of microRNAs were found closely related to tumor development, such as miR-30b [25], miR-155 [26], and miR-16 [27, 28]. miR-30b has been shown to inhibit tumor cell proliferation, invasion, and metastasis [25]. miR-155 was also reported to be a tumor suppressor activated via the targeting of many key proteins [26]. Together with previous reports, the data gathered in this report indicates that SeNP-regulated microRNAs likely play an important role in mediating SeNP functions. Among the identified miRNAs, miR-16 was measured as the most dramatically different between the SeNP treatment group and the control group. miR-16 has been reported as highly related to cell cycle and apoptosis regulation [27, 28]. These results demonstrated that SeNPs upregulate miR-16, therefore downregulating two key targets of miR-16: cyclin D1 and BCL-2. This may be the direct route through which SeNPs promote cancer cell apoptosis. The miR-16 level in seven other tumor cell lines, including Colon-26, HepG2, Hela, A549, MCF7, A498, and WM-115, was measured, and it was found that SeNPs can also upregulate miR-16 expression in these cells (data not shown). Considering that miR-16 can regulate tumor development in a variety of tumor types [29], we conclude that the miR-16 upregulation by SeNPs may be a common anticancer mechanism in a variety of tumor cells.

SeNP antitumor effects against CaP led us to consider whether selenium levels in patients could affect tumor prognoses. A positive correlation between serum selenium levels and miR-16 expression was found. Furthermore, higher selenium concentrations were associated with better prognoses, confirming the trends measured in the in vitro assays of this study.

One limitation of this article is that the activity of SeNPs against CaP in animal models has yet to be confirmed. However, this work is already underway. The goal of these follow-up animal experiments is to test the toxicity and pharmacokinetic profile of SeNPs. Some earlier publications offer inspiration in this direction of study: Shahverdi et al. found that SeNPs had low toxicity and potent activity in a mouse model of breast cancer [30]. Meanwhile, Nazıroğlu et al. summarized their recent in vivo studies on animals treated with intraperitoneal or oral nanoparticles, where they found that nanoparticles, including SeNPs, can accumulate around tumors and cause direct cytotoxic effects on tumor cells [31]. These results encourage us to further explore the cytotoxic effects of SeNPs against CaP in vivo. Another limitation of this study is that it cannot be proven conclusively whether SeNPs only work through miR-16, or whether other mechanisms in mediating SeNPs functions and future work on the application of selenium nanoparticles, are to be expected.

## Conclusion

Taken together, these results have evidently signified that SeNPs have strong killing activity against CaP cells, and high selenium content in CaP patients may indicate a better prognosis.

## Data Availability

The datasets used and/or analyzed during the current study are available from the corresponding author on reasonable request.
